# Fecal Microbial Transplantation versus Mesalamine Enema for Treatment of Active Left-Sided Ulcerative Colitis—Results of a Randomized Controlled Trial

**DOI:** 10.3390/jcm10132753

**Published:** 2021-06-22

**Authors:** Jan Březina, Lukáš Bajer, Pavel Wohl, Dana Ďuricová, Pavel Hrabák, Aleš Novotný, Jana Koželuhová, Milan Lukáš, Jakub Mrázek, Kateřina Olša Fliegerová, Simona Kvasnová, Mekadim Chahrazed, Jan Mareš, Julius Špičák, Pavel Drastich

**Affiliations:** 1 Hepatogastroenterology Department, Institute for Clinical and Experimental Medicine, 14021 Prague, Czech Republic; bajl@ikem.cz (L.B.); pawo@ikem.cz (P.W.); janmares42@gmail.com (J.M.); jusp@ikem.cz (J.Š.); padr@ikem.cz (P.D.); 2 Clinical and Research Centre for IBD ISCARE, 19000 Prague, Czech Republic; duricova@iscare.cz (D.Ď.); milan.lukas@email.cz (M.L.); 3 4th Department of Medicine—Department of Gastroenterology and Hepatology, First Faculty of Medicine, Charles University and General University Hospital in Prague, 12808 Prague, Czech Republic; pavel.hrabak@vfn.cz (P.H.); novotnyaales@seznam.cz (A.N.); 4 Gastroenterology Department, I. Internal Clinic, University Hospital in Pilsen, 30100 Pilsen, Czech Republic; kozeluhova@fnplzen.cz; 5 Institute of Animal Physiology and Genetics of the Czech Academy of Science, v.v.i., 14220 Prague, Czech Republic; mrazek@iapg.cas.cz (J.M.); fliegerova@iapg.cas.cz (K.O.F.); kvasnova@iapg.cas.cz (S.K.); mekadim@iapg.cas.cz (M.C.)

**Keywords:** ulcerative colitis, fecal microbial transplantation, 5-aminosalicylic acid

## Abstract

Background and Aims: Ulcerative colitis (UC) is a chronic inflammatory disease. Fecal microbial transplantation (FMT) is a promising alternative treatment. Methods: This multicenter, open-label, noninferiority trial randomized patients with active left-sided UC (Mayo score 4–10) equally to FMT or 5-aminosalicylic acid (5-ASA) enemas. FMT enemas were administered five times in the first week and then once weekly for 5 weeks. 5-ASA enemas were administered daily for 2 weeks and then every other day. The primary study endpoint was clinical remission, with a total Mayo score ≤2 at week 12 with no subscore >1. Results: Sixty-one patients were screened; 45 were enrolled and randomized to FMT (*n* = 23) or 5-ASA (*n* = 22). Twenty-one FMT and 22 5-ASA patients completed at least the week 4 study visit and were included in the mITT analysis. Twelve FMT (57%) and eight 5-ASA patients achieved the primary study endpoint. FMT noninferiority with 10% margin was confirmed (95% CI: −7.6%, 48.9%). Adverse events occurred in 12 FMT (57%) and 13 5-ASA (59%) patients. Increased microbial diversity persisted 3 months after FMT. Conclusion: FMT is an effective treatment for left-sided UC and increased recipient microbiome diversity. Targeted microbiome modification may improve FMT efficacy. Further investigation is needed to guide donor and patient selection.

## 1. Introduction

Ulcerative colitis (UC) is a chronic inflammatory disease of the colonic mucosa and submucosa. Despite significant improvements in therapy, which include biological treatment, some patients continue having disease manifestations such as bloody diarrhea and cramps, as well as persistent colonic inflammation, while others experience adverse effects of medical therapy of varying severity. The pathogenesis of UC is not yet fully understood, but it is thought to involve failure to maintain immune and microbiome homeostasis. Compared with the general population, the gut microbiome in patients with UC is significantly less diverse and less stable over time [1,2]. It has not been established whether that is a cause or a consequence of UC. The benefits of prebiotics, probiotics, and synbiotics in the treatment of UC are inconclusive [3,4]. Presumably, the most effective way to change the composition of the gut microbiome is fecal microbial transplantation (FMT). For example, FMT has shown excellent efficacy in recurrent *Clostridoides difficile* infection [5,6]; so far, five randomized controlled trials (RTCs) have reported promising results of FMT in the treatment of UC [7,8,9,10,11]. Three of four studies (Moayyedi et al., Paramsothy et al., and Costello et al.) examining FMT in inducing remission in active UC demonstrated FMT superiority over placebo [7,9,10]. However, differences in the methods and frequencies of administration and evaluation criteria do not allow clear conclusions to be drawn. None of the studies specifically evaluated patients with left-sided UC, which accounts for 30–50% of the cases [12]. Even though their clinical course and therapy may differ, left-sided colitis is perceived as the same disease as pancolitis [12]. In left-sided colitis, topical mesalamine is superior to oral therapies and topical steroids, and it has an excellent safety profile; therefore, it is considered a standard of care [13]. As FMT administered as an enema can be seen as a topical therapy, we decided to compare its efficacy and safety with that of mesalamine in the noninferiority FACTU (fecal bacteriotherapy for ulcerative colitis) trial, the first randomized controlled trial (RCT) to compare FMT enema with topical 5-aminosalicylic acid (5-ASA) therapy in patients with left-sided UC. For feasibility reasons, we decided for a noninferiority trial with the aim of paving the way for a possible larger trial in the future, which could even show the superiority of FMT to mesalamine.

## 2. Materials and Methods

### 2.1. Study Design

This was an open-label randomized noninferiority trial comparing the treatment efficacy of FMT enema and standard 5-ASA enema in patients with active left-sided UC. The patients were enrolled in four inflammatory bowel disease (IBD) centers in the Czech Republic. They were the Institute for Clinical and Experimental Medicine (IKEM, Prague, Czechia), Clinical and Research Centre for IBD (ISCARE, Prague, Czechia), General University Hospital in Prague, and University Hospital in Pilsen. The local research ethics committees at each site approved the trial, and the Ministry of Health of the Czech Republic authorized the use of FMT in the treatment of left-sided UC for this study. All participants gave their signed informed consent. The trial was registered on ClinicalTrials.gov, number NCT03104036 (https://clinicaltrials.gov/ct2/show/NCT03104036, accessed on 25 January 2021).

### 2.2. Study Population

We enrolled adult patients younger than 70 years of age with clinically and endoscopically active left-sided UC (i.e., extent of more than 15 cm and less or to the lienal flexure) of >3 months duration, a total Mayo score of 4–10, and endoscopy subscore ≥2. Use of oral 5-aminosalicylates (stable dose for 8 weeks, maximal dose 4 g), thiopurines (stable dose for 8 weeks), and oral prednisone (≤10 mg daily and stable for 4 weeks) was allowed. After inclusion, patients had to remain on the same dosage during the study period. The exclusion criteria are shown in detail in Table S1 (Supplementary Materials). Briefly, individuals with indeterminate colitis, Crohn’s disease, irritable bowel syndrome, a history of bowel cancer, and positivity for *Clostridoides difficile* infection or another enteric pathogen, as well as pregnant or breastfeeding women, were excluded. Use of rectal corticosteroids or 5-aminosalicylate in the 4 weeks before enrolment, antibiotics or probiotics in the 8 weeks before enrolment, methotrexate in the 8 weeks before enrolment, or biological therapies or calcineurin inhibitors in the 12 weeks before enrolment was not allowed. At study entry, all potentially eligible patients underwent colonoscopy or sigmoidoscopy. We excluded those with gastrointestinal infections including bacterial and parasitic pathogens, cytomegalovirus, and *Clostridoides difficile*.

### 2.3. Randomization

Eligible patients were randomized 1:1 using a computer-generated randomization list stratified by gender and the receipt of immunosuppressive therapy. The randomization was performed centrally at IKEM in Prague.

### 2.4. Interventions

We administered 10 study FMT infusions to the treatment group, five in the first week and once weekly in the following 5 weeks. Participants in the 5-ASA group were treated with a standard-of-care regimen that included 4 g mesalamine enemas daily for 2 weeks and then every other day until the end of week 6. Enema tolerance was defined as retaining the enema for at least 15 min.

### 2.5. Clinical Outcomes

The primary study endpoint was clinical remission, which was defined as a total Mayo score ≤2 with no subscore >1 at week 12. Secondary endpoints were clinical response, defined as a reduction in total Mayo score ≥2 points, and endoscopic remission, defined as an endoscopic Mayo score of 0 at weeks 6 and 12. Endoscopic disease activity was assessed by sigmoidoscopy at weeks 6 and 12, and the total Mayo score was calculated. Endoscopies were performed and recorded at each study center and were then centrally assessed by two endoscopists blinded to the administered therapy. Any discrepancies were resolved by discussion and agreement. Adverse events were assessed at every study visit and reported following the Common Terminology Criteria for Adverse Events, version 5.0.

### 2.6. FMT Enema Preparation

Fecal samples were collected at each center from healthy donors who were younger than 60 years of age. We excluded blood relatives, individuals hospitalized in the previous 3 months or treated with antibiotics or proton pump inhibitors in the previous 6 months, immunosuppressed individuals, and those with a history of chronic gastrointestinal tract problems (e.g., IBD, constipation, functional dyspepsia), autoimmune diseases, or obesity. Donors with a history of travel to risk areas in the previous 3 months were also excluded. All donors underwent rigorous screening for infectious diseases (Table S2, Supplementary Materials). Each study center was responsible for potential donor selection. Each stool sample was analyzed by 16S rRNA sequencing; at each center, a donor with the greatest microbiome diversity and an alternate were selected. We then used those donors for all the patients participating at a single center. Each patient received FMT from the same donor over the entire study.

Donor stool samples were evaluated by study staff for the presence of mucus and blood and assessed for consistency. The sample was weighed, 150 mL of saline was added for each 50 g of stool, and the suspension was homogenized with a standard kitchen mixer. The resulting product was filtered twice through sterile gauze to remove large stool residues. Glycerol was added as a cryoprotectant, and the sample was transferred to study containers. The volume of each infusion was 150–170 mL. The infusions were stored at −80 °C. On the day of administration, the study infusions were thawed for 1 h at normal room temperature and then for 1 h in a 37 °C bath. The study infusions were administered immediately after thawing to the patients at the study site.

### 2.7. Assessment and Analysis of the Microbiome

Fecal samples were collected at baseline and each study visit at weeks 2, 4, 6, and 12 in the FMT group, at weeks 2 and 12 in the 5-ASA group, and at the 1 year follow-up in all patients, if completed. Stool samples from patients and donors were frozen and stored at −80 °C for further analysis. Genomic DNA was extracted from frozen samples using Power Fecal DNA isolation kits (QIAGEN, Hilden, Germany) with bead-beating cell disruption in a FastPrep-24 homogenizer (MP Biomedicals, Irvine, CA, USA). The isolated DNA was checked with a NanoDrop 2000c UV/Vis spectrophotometer (Thermo Scientific, Waltham, MA, USA) and stored at −20 °C until use. The DNA was used to prepare V4-5 16S rDNA amplicons as described by Fliegerová et al. [14]. The amplicons were purified with a Monarch DNA Clean up kit (New England Biolabs, Ipswich, MA, USA) and used for high-throughput sequencing on an Ion Torrent platform following the manufacturer’s protocol.

Bacterial 16S rRNA gene sequences were obtained in FASTQ format for analysis with QIIME 2 2020.2 pipelines [15]. For sequence quality, DADA2 was used as a noise filter and to remove chimeric sequences [16]. VSEARCH was used for clustering and taxonomy classification of the filtered sequences using the SILVA database [17]. The Shannon index of diversity and principal coordinate analysis (PCoA) of Bray–Curtis distance were assessed after the samples were rarefied to 5000 sequences each. Linear discriminant analysis (LDA) with an effect size (LefSe) algorithm [18] in the Galaxy Web module (http://huttenhower.sph.harvard.edu/galaxy/, accessed on 24 March 2021) was performed for biomarker identification. The factorial Kruskal–Wallis and pairwise Wilcoxon tests were used to detect taxa with significant differential relative abundance of bacterial families in treatment responders and non-responders. The alpha value was 0.05, with a threshold value of 2.0 for the logarithmic LDA scores of discriminative features.

### 2.8. Statistical Analysis

We aimed to recruit 50 patients with active UC (25 in each arm), based on a sample-size calculation that assumed a 30% remission rate in the 5-ASA arm [19] and a 60% remission rate in the FMT arm [20], requiring 80% power in a noninferiority design with a 95% two-sided confidence interval (CI) and a 10% noninferiority margin, and assuming a 5% attrition rate. Descriptive statistics (median, maximum, minimum values) were used to describe the results. The 95% CI of the differences in treatment success rate was calculated by bootstrapping with 10⁶ iterations. For the secondary outcomes, differences between treatments were tested by Fisher‘s exact test. The analysis was performed in a modified intention-to-treat (mITT) population requiring tolerance of the treatment for inclusion. We used Python 3.7.4 (numpy 1.16.5, scipy 1.3.1, matplotlib 3.1.1) for the statistical analysis.

## 3. Results

**Figure 1:**
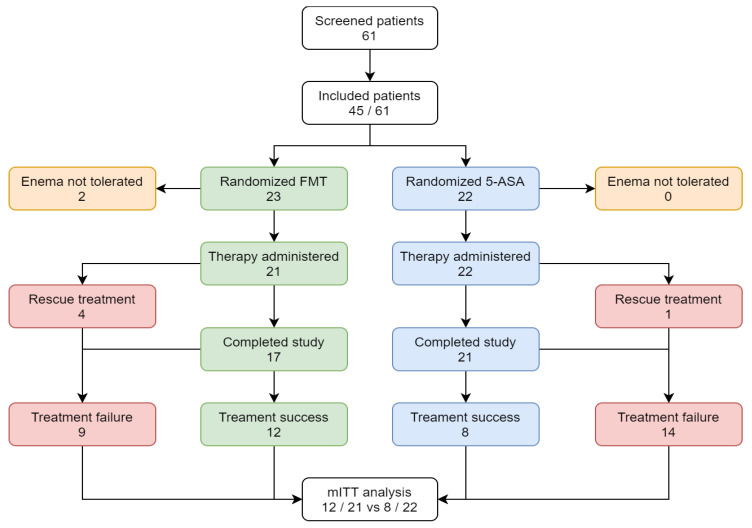
Trial profile. 5-ASA, 5-aminosalicylic acid; FMT, fecal microbial transplant; mITT, modified intention-to-treat.

Sixty-one patients were recruited between April 2017 and October 2020. After screening, 45 were randomly allocated to either FMT (*n* = 23) or 5-ASA enema (*n* = 22). Two patients in the FMT group who did not tolerate the first enema were not included in the final analysis. Forty-three patients, 21 in the FMT group and 22 in the 5-ASA group, completed at least the visit in week 4 and were included in mITT analysis (Figure 1). The baseline demographic and clinical characteristics of both groups were similar except for a longer disease duration in the FMT group, a difference that was not significant (*p* = 0.07, Table 1).

### 3.1. Clinical Outcome

Table 2 presents the outcome results at weeks 6 and 12. Twelve of the 21 FMT patients (57%) and eight of the 22 5-ASA enema patients (36%) successfully achieved the primary study endpoint at week 12. The noninferiority of FMT with 10% margin was confirmed (95% CI: −7.6%, 48.9%. Only one responder in each group was on corticosteroid therapy at week 12. No statistically significant between-group differences in the secondary outcomes were found. A clinical response was achieved at week 6 by 14 FMT patients (64%) and 12 5-ASA patients (55%, *p* = 0.53) and at week 12 by 15 FMT patients (71%) and 12 5-ASA patients (55%, *p* = 0.35). Endoscopic remission was achieved at week 6 by three FMT patients (14%) and one 5-ASA patient (5%), *p* = 0.34) and at week 12 by three FMT patients (14%) and three 5-ASA patients (14%, *p* = 1.0).

### 3.2. Safety

Twelve FMT patients (57%) and 13 5-ASA patients (59%) had at least one adverse event during the 12 week period of study observation. Between-group differences in the number or type of adverse events were not significant (Table 3). The most common adverse events were self-limiting gastrointestinal complaints. Five serious adverse events occurred during study treatment, four in patients with FMTs and one in a patient given 5-ASA enemas. In all cases, it was a worsening of colitis with the need for treatment intensification (i.e., an increase in oral corticoids in two cases, intravenous corticoids in one case, and biologic therapy in two cases). No patient required a colectomy during the study period or during the 1 year follow-up, if completed. Enema tolerance was generally good, with only two patients in the FMT group and none in the 5-ASA group experiencing intolerance.

### 3.3. Donor Selection

Eleven donors from the four centers were tested for the most suitable microbiota composition. Following evaluation of relative taxonomies (Figure S1, Supplementary Materials) and calculation of diversity indices, four donors were chosen, one at each medical center. Phylum Firmicutes was predominant in all stool samples with a prevalence of families Lachnospiraceae (50.3–70.3%) and Ruminococcaceae (12.6–23%). The genera of butyrate-producing bacteria included *Blautia*, *Roseburia*, and *Faecalibacterium*.

### 3.4. Microbiome Outcomes

**Figure 2:**
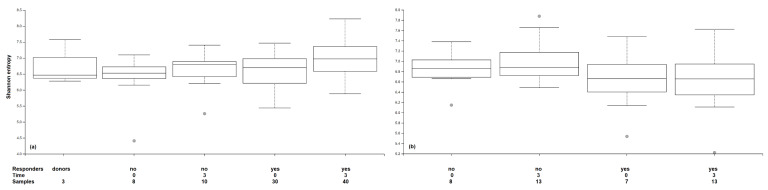
Microbial diversity expressed as Shannon entropy index of treatment responders and non-responders for (**a**) 5-aminosalicylates group and (**b**) fecal microbial transplantation group and the donors. The samples are grouped based on responsiveness to the treatment (yes/no) and time point of a sample collection 0 (before therapy) and 3 (weeks 1–12), the circle symbol represents outliers.

**Figure 3:**
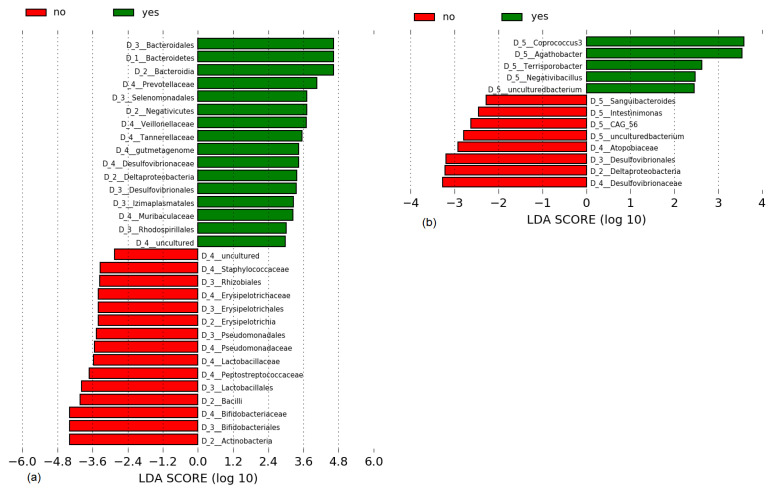
Linear discriminant analysis (LDA) scores of the microbial taxa of responders (yes) and non-responders (no) to (**a**) fecal microbial transplantation and (**b**) 5-aminosalicylic acid treatment of patients with left-sided ulcerative colitis.

The microbiome results obtained in the 3 months following treatment are reported here. The 1 year results are not available at this time because of study delays and will be published later. A total of 135 fecal samples from 35 patients were analyzed by high-throughput sequencing. Forty-seven samples were from 17 patients given 5-ASA enemas and 88 were from 18 patients with FMTs. A total of 13 phyla were detected in the stool samples from patients with UC in the FMT group. Two dominant phyla were found in all samples, Firmicutes (54.4–67.7%) and Bacteroidetes (15.5–27.1%), which were represented mainly by orders Clostridiales and Bacteroidales. The predominant Actinobacteria (7.6–16.6%) were Bifidobacteriales and Coriobacteriales. The predominant Proteobacteria (1.8–6%) were Gammaproteobacteria. The relative abundance of other phyla including Fusobacteria, Lentisphaerae, Verrucomicrobia, Tenericutes, Patescibacteria, Epsilonbacteraeota, Cyanobacteria, Cloacimonetes and Acidobacteria was very low (all ≤ 0.5%). Three months after FMT treatment, decreases in the relative abundance of order Bacteroidales and family Bacteroidaceae were detected in the responders. The FMT responders showed partial clustering of microbiota after treatment and increased diversity indices (Figure 2). That was confirmed by LDA, which revealed 31 taxa with significantly different abundance (LDA scores > 2) in responders and non-responders (Figure 3). Bacteroidales, Prevotellaceae, Veillonellaceae and Desulfobacteria were significantly higher in responders. Staphylococcaceae, Lactobacillaceae and Bifidobacteriaceae were significantly higher in non-responders. Lastly, PCoA of the Bray–Curtis distance matrix revealed a high clustering power of samples of personal origin, with only a minor contribution from FMT responders versus non-responders (Figure S2, Supplementary Materials).

**Figure 4:**
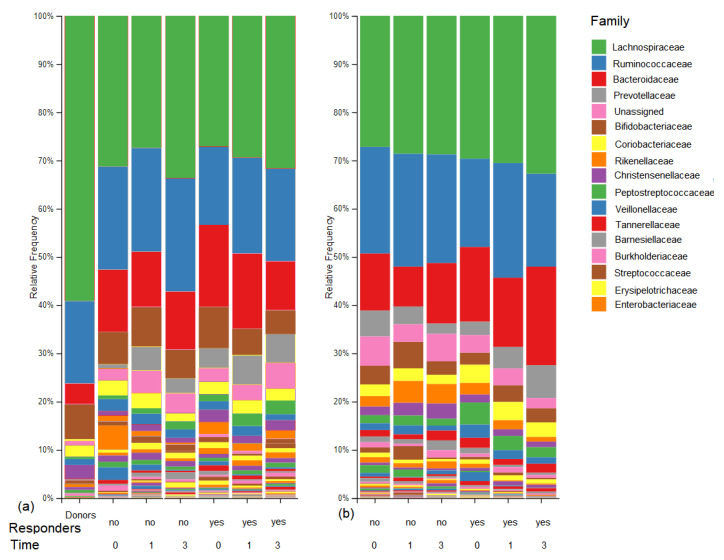
Relative abundance of fecal bacteria at the family level in all 135 samples from 35 patients with left-sided ulcerative colitis treated by (**a**) fecal microbial transplantation or (**b**) aminosalicylic acid. Donor profiles are shown in (**a**). The results are grouped by responsiveness to the treatment (yes/no) and the time of collection: 0 (before treatment), 1 (2–4 weeks after treatment), and 3 (12 weeks after treatment).

A total of eight phyla were identified in the samples of patients with UC in the 5-ASA group. Firmicutes (59.3–65.8%) and Bacteroidetes (19–30.7%) were the dominant phyla in all samples regardless of the time of sampling. Actinobacteria (5.7–9.1%), Proteobacteria (1–4.1%), and Tenericutes (0.1–2.1%) were present in all samples. Phylum Firmicutes was mainly represented by members of order Clostridiales, phylum Bacteroidetes was mainly represented by order Bacteroidales, and order Bifidobacteriales was the predominant representative of the phylum Actinobacteria. Other phyla, including Fusobacteria, Lentisphaerae, and Verrucomicrobia were detected at low frequencies (all ≤ 0.5%). Three months after treatment, increased relative abundance of order Bacteroidales, primarily families Bacteroidaceae and Prevotellaceae, was still detected in the 5-ASA responders (Figure 4).

Significant changes in the microbial taxa after 5-ASA treatment were not seen in the patients who were tested (Figure 4). However, linear LDA effect size revealed differences in the relative abundance of 13 taxa between responders and non-responders (Figure 3). The main differences were the enhanced presence of the genera *Coprococcus* and *Agathobacter* in responders and family Desulfovibrionaceae and class Deltaproteobacteria in non-responders.

## 4. Discussion

This study assessed the clinical efficacy of FMT enema in patients with left-sided UC with mild to moderate disease activity. Our results provide evidence that FMT enemas were not clinically inferior to standard care with 5-ASA enemas. More than half of the FMT patients experienced clinical remission at week 12. Differences in the secondary outcomes of clinical response and endoscopic remission achieved in the FMT group and the 5-ASA enema comparator group were not significant. Treatment with FMT enemas had a good safety profile and was generally well tolerated. We observed increased gut microbial diversity in both groups; however, over 3 months of follow-up, FMT sustained an effect compared to 5-ASA group.

Five RCTs have been published to date on the use of FMT in UC. Four evaluated the achievement of remission in active UC [7,8,9,10] and one evaluated the maintenance of remission [11]. Two were published in 2015 and were terminated early for presumed lack of effectiveness [7,8]. However, in one of those studies by Moayyedi et al., a final analysis found that remission, with a full Mayo score of ˂3, was achieved at week 7 by 25% of the participants after receiving once-weekly FMT enemas for 6 weeks [7]. A subsequent study by Paramsothy et al. found that a very intensive FMT regimen including a single colonoscopic administration followed by enemas given 5 days per week for 8 weeks achieved a steroid-free clinical remission rate of 27% compared with 8% for placebo [9]. Similar results were obtained by an Australian study that reported a steroid-free remission of 32%, despite a much less intensive treatment regimen of one colonoscopic infusion followed by two enemas over 7 days [10]. In our study, 57% of the patients treated with FMT enemas achieved clinical remission at week 12. The remission rate is higher than that reported in previous studies, and it does not appear to be affected by corticoid use, which was minimal in our study. Only one FMT patient using corticosteroids achieved clinical remission, for a hypothetical steroid-free clinical remission rate of 52%. The results may have been influenced by our study population, which included only patients with mild to moderately active UC compared with moderate to severe UC in previous studies. We also believe that the patients in our FMT group benefited from the intervention regimen, in which five consecutive administrations in the first week and five enemas in each of the following 5 weeks promoted and sustained the microbiome changes.

Key features distinguishing our trial from previous studies of FMT in UC were donor selection by 16S rRNA sequencing and the focus on left-sided UC. Previous studies have reported that FMT using material from donors with high microbiome diversity was associated with improved clinical outcomes in the treatment of UC [7]. Donor prescreening to select those with the highest microbiome diversity could improve the effectiveness of FMT, but this has to be verified in future studies.

Previous studies of the efficacy of FMT for the treatment of UC did not take into account the extent of UC. By including patients with pancolitis, left-sided colitis, and proctitis, the topical effect of FMT might be overlooked. UC was characterized by Moayyedi et al. as a disorder that originates in the rectum, with the rectum as the site of most dysbiosis [21]. Pancolitis in *Clostridoides difficile* infection has been effectively treated by FMT retention enemas [22], but UC is characterized by a complex interaction of genetics, microbiome, and environment that might result in increased resistance to the therapeutic effect of FMT in extensive disease [23]. Therefore, we hypothesized that focusing on left-sided UC might lead to better treatment efficacy. Further study comparing FMT efficacy in disease that varies in extent is needed for clarification.

Our study was designed to test the noninferiority of FMT to 5-ASA enema for the treatment of UC. FMT has already been shown to be superior to placebo for the treatment of UC [7,9,10]. That was an important step toward achieving its clinical application, but comparison to other available treatments is necessary. As a superiority trial would require a number of patients beyond the capabilities of our four centers, we opted for the noninferiority design. First, proof on noninferiority allows applying an alternative treatment with some other beneficial properties different from the treatment success. Second, as FMT tends to be actually superior to 5-ASA in treatment success, our study makes the starting point easier for a future superiority trial. The potential future patients are not put at risk of receiving a potentially highly inferior treatment. Only one responder in each group was on corticosteroid therapy at week 12.

Recently, it was reported that the diversity, composition, and bacterial interaction patterns in mucosal samples of patients with UC were altered after 5-ASA treatment [24]. In this study, Shannon’s diversity index of fecal microbiota was lower in patients with UC before FMT treatment and in non-responders than it was in healthy donors. The diversity index increased in the patients who responded to FMT, and the microbiota composition changed to resemble that seen in a healthy donor. Similar findings were discussed by Khanna et al. in a review published in 2017 [25].

In this study, the relative abundance of Lachnospirceae and Ruminococcaceae increased gradually and that of Bacteroidaceae decreased gradually, becoming similar to the abundance in healthy donors. The opposite was noticed in the non-responder patients. An increase in family Lachnospiraceae was also observed in our previous study [26]. At the genus level, *Blautia* and *Fecalibacterium* increased and *Bacteroides* decreased after FMT treatment, but their abundance remained different from that in healthy donors. Consistent with our finding, a significant increase of *F. prausnitzii* after FMT was reported in patients with mild to moderately active UC [27]. Similar to our findings, in a study of FMT in patients with *Clostridoides difficile* infection, the relative abundance of *Faecalibacterium* was significantly increased in those with IBD and that of *Blautia* was increased in those without IBD. After FMT, the abundance of *Bacteroides* was increased in patients with IBD [25].

In this study, LEfSe analysis indicated that there were differences in the intestinal microbiota between responders and non-responder patients after FMT treatment. Sokol et al. suggested that the success of FMT therapy and donor microbiota colonization might be affected by the recipient’s baseline characteristics [28]. In our patients, FMT enemas from all four donors had positive effects on the recipients’ microbiota and host health despite differences in the pretreatment bacterial profiles. 5-ASA treatment caused an increase in Firmicutes and Actinobacteria phyla and a decrease in *Proteobacteria* 1 month after treatment, which was also observed by Olaisen et al. in 2019 [29]. However ongoing monitoring found that the changes in microbiota composition were reversed by 3 months, suggesting that the original core mucosa was able to restore its original composition. In our FMT patients, the increase in Firmicutes, mainly family Lachnospiraceae, and decrease in Bacteroidaceae and Enterobacteriaceae were maintained at 3 months after treatment. The persistence of a microbiota shift toward the donor composition is in agreement with previous studies that tested long-term fecal microbiota transplantation in patients with *Clostridoides difficile* infection [30] and in healthy volunteers [31]. However, this is the first study to report the benefits of FMT in patients with UC. LDA of pretreatment microbiota did not identify any microbial species associated with patient responsiveness to either 5-ASA or FMT.

Our study has some limitations. The main limitation is its open-label design, which might have led to overestimation of clinical remission and FMT response rates because of the placebo effect. However, in a recent Australian trial of FMT in patients with active UC, efficacy did not differ between the blinded and open-label arms [9]. Another drawback was the inability to enroll the planned number of study participants because recruitment was limited by the small number of participating centers and the discontinuation of study funding. Despite these limitations, we achieved the primary endpoint. A minor limitation was the reduced number of stool samples included in the microbial analysis because of storage problems at one study center.

In conclusion, our study evidence supports FMT enema as a promising treatment of left-sided UC, which is associated with a significant increase in microbiome diversity. Targeted microbiome modification may contribute to increased FMT efficacy, with potential as a novel option for difficult-to-treat UC. Further research is needed to identify suitable donors and patients for FMT and to clarify long-term outcomes.

## Figures and Tables

**Table 1:** Patient baseline characteristics.

	Fecal Microbiota Transplantation (*n* = 23)	5–ASA Enema (*n* = 22)
Male	12 (52%)	11 (50%)
Female	11 (48%)	11 (50%)
Age	39 (25–63)	39.5 (27–70)
Disease duration (years)	9 (1–20)	4.5 (0.6–20)
Prior biologic exposure	0 (0%)	0 (0%)
Concomitant therapy		
Oral 5-aminosalicylate	19 (83%)	18 (82%)
Oral steroids	3 (13%)	2 (9%)
Oral immunomodulator	5 (22%)	4 (18%)
Total Mayo score	6 (4–10)	6 (4–10)
Endoscopic Mayo = 2	19 (83%)	18 (82%)
Mayo = 3	4 (17%)	4 (18%)
Fecal calprotectin (µg/g)	1817.5 (166–6000)	1220 (80–6000)
C-reactive protein (mg/L)	2.3 (0.3–25)	2.1 (0.4–32.8)
White-cell count (×10⁹ cells/L)	7.9 (5.4–11.7)	6.3 (3.9–12.3)
Hemoglobin (g/L)	141 (107–163)	142 (104–161)

Data are the number of patients (%) or median (range). 5-ASA, 5-aminosalicylic acid.

**Table 2:** Primary and secondary outcomes at weeks 6 and 12.

	Fecal Microbiota Transplantation (*n* = 21)	5-ASA Enema (*n* = 22)	95% CI for Difference
**Primary outcome**			
Clinical remission (week 12)	12 (57%)	8 (36%)	(−7.6%, 48.9%)
**Secondary outcome**			***p*-value**
Clinical response (week 6)	14 (64%)	12 (55%)	0.53
Clinical response (week 12)	15 (71%)	12 (55%)	0.35
Endoscopic remission (week 6)	3 (14%)	1 (5%)	0.34
Endoscopic remission (week 12)	3 (14%)	3 (14%)	1.0

5-ASA, 5-aminosalicylic acid; CI, confidence interval.

**Table 3:** Adverse events.

	Fecal Microbiota Transplantation (*n* = 21)	5-ASA Enema (*n* = 22)
Total adverse events	22	21
Total patients with adverse events	12 (57%)	13 (59%)
Total patients with serious adverse events	4 (19%)	1 (5%)
Infection	1 (5%)	0 (0%)
Worsening of ulcerative colitis	8	9
Abdominal pain	5	8
Bloating	2	1
Rash	0	1
Fever	2	1

5-ASA, 5-aminosalicylic acid.

## Data Availability

The data underlying this article can be shared on reasonable request to the corresponding author. The data are not publicly available due to privacy reasons.

## Supplementary Materials

The following are available online at https://www.mdpi.com/article/10.3390/jcm10132753/s1: Figure S1. Relative abundance of common bacterial families of stool donors; Principal coordinate analysis showing Bray Curtis distance matrix between patients treated by (a) aminosalicylates and (b) faecal microbial transplantation; Table S1. Study exclusion criteria; Table S2. Donor examination.
