# Supplementary material for: Fecal Microbial Transplantation versus Mesalamine Enema for Treatment of Active Left-Sided Ulcerative Colitis—Results of a Randomized Controlled Trial
Source: J Clin Med. 2021 Jun 22;10(13):2753. doi: 10.3390/jcm10132753 (PMC8268406; doi:10.3390/jcm10132753)
Supplement: Supplementary file 1 [file jcm-10-02753-s001.zip › Figure S2.pdf]

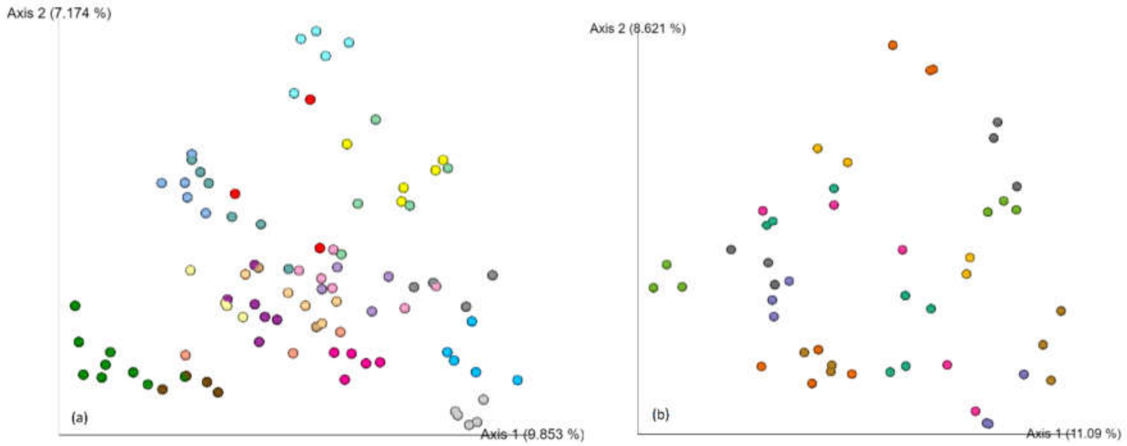

Figure S2. Principal coordinate analysis showing Bray Curtis distance matrix between patients treated by (**a**) aminosalicylates and (**b**) faecal microbial transplantation. Stool donors are showed as red dots, other colours represent patients.
