# Supplementary material for: Fecal Microbial Transplantation versus Mesalamine Enema for Treatment of Active Left-Sided Ulcerative Colitis—Results of a Randomized Controlled Trial
Source: J Clin Med. 2021 Jun 22;10(13):2753. doi: 10.3390/jcm10132753 (PMC8268406; doi:10.3390/jcm10132753)
Supplement: Supplementary file 1 [file jcm-10-02753-s001.zip › Table S1.pdf]

Table S1. Study exclusion criteria.

Anti-TNF medication and other biologic therapy in the previous 12 weeks
Rectal corticosteroids or 5-aminosalicylate in the previous 4 weeks
Calcineurin inhibitors in the previous 12 weeks
Methotrexate in the previous 8 weeks
Prednisone > 10mg
Antibiotics or probiotics in the previous 8 weeks
The real risk of colectomy in the near future
Positive stool culture (*Salmonella*, *Shigella*, *Yersinia*, *Campylobacter*, pathogenic *Escherichia coli*)
Cytomegalovirus infection, *Clostridium difficile* infection
Pregnancy, breastfeeding women
Indeterminate colitis, Crohn's disease, irritable bowel syndrome
History of bowel cancer
Age > 70 years
