# Supplementary material for: Fecal Microbial Transplantation versus Mesalamine Enema for Treatment of Active Left-Sided Ulcerative Colitis—Results of a Randomized Controlled Trial
Source: J Clin Med. 2021 Jun 22;10(13):2753. doi: 10.3390/jcm10132753 (PMC8268406; doi:10.3390/jcm10132753)
Supplement: Supplementary file 1 [file jcm-10-02753-s001.zip › Table S2.pdf]

Table S2. Donor examination.

**Blood test: hepatitis A, B, C, HIV-1, HIV-2, *Treponema pallidum***
**Blood test for active infection: cytomegalovirus, Epstein-Barr, herpes simplex, Varicella zoster**
**Stool tests: *Yersinia spp.*, *Salmonella spp.*, *Shigella spp.*, *Campylobacter jejuni*, *Clostridium difficile* toxin, *Helicobacter pylori***
**Parasitological examination**
